# Key Role of Staphylococcal Fibronectin-Binding Proteins During the Initial Stage of *Staphylococcus aureus* Keratitis in Humans

**DOI:** 10.3389/fcimb.2021.745659

**Published:** 2021-11-09

**Authors:** Corantin Maurin, Emilie Courrier, Zhiguo He, Josselin Rigaill, Jérôme Josse, Frédéric Laurent, Philippe Gain, Gilles Thuret, Paul O. Verhoeven

**Affiliations:** ^1^ Corneal Graft Biology, Engineering and Imaging Laboratory (BiiGC), University of St-Etienne, St-Etienne, France; ^2^ CIRI, Centre International de Recherche en Infectiologie, GIMAP Team, University of Lyon, University of St-Etienne, INSERM U1111, CNRS UMR5308, ENS de Lyon, UCBL1, St-Etienne, France; ^3^ Laboratory of Infectious Agents and Hygiene, University Hospital of St-Etienne, St-Etienne, France; ^4^ CIRI, Centre International de Recherche en Infectiologie, Staphylococcal Pathogenesis Team, University of Lyon, INSERM U1111, CNRS UMR5308, ENS de Lyon, UCBL1, Lyon, France; ^5^ Department of Bacteriology, Institute for Infectious Agents, Hospices Civiles de Lyon, Lyon, France; ^6^ Centre National de Référence des Staphylocoques, Hospices Civils de Lyon, Lyon, France; ^7^ Department of Ophthalmology, University Hospital, St-Etienne, France

**Keywords:** *Staphylococcus aureus*, keratitis, fibronectin (FN), adhesion, internalization, MSCRAMMs, human cornea, primary human cells

## Abstract

**Objectives:**

*Staphylococcus aureus* is one of the main causes of bacterial keratitis in humans. This study was aimed at investigating the mechanisms of *S. aureus* adhesion to the human corneal epithelium involved during the initial stage of infectious keratitis.

**Methods:**

Human corneas stored in a specific active storage machine that restores a normal pluristratified epithelium were used to assess *S. aureus* adhesion level to intact and injured tissues using immunostaining. *S. aureus* adhesion to immobilized fibronectin was measured in microtiter plate. Internalization of *S. aureus* clinical isolates recovered from keratitis was assessed on human corneal epithelial HCE-2 cells.

**Results:**

Superficial corneal injury unmasked fibronectin molecules expressed within the human corneal epithelium. *S. aureus* adhesion level was increased by 117-fold in the area of injured epithelium (*p* < 0.0001). The deletion of staphylococcal *fnbA/B* genes decreased by 71% the adhesion level to immobilized fibronectin (*p* < 0.001). The deletion of *fnbA/B* genes and the incubation of the corneas with anti-fibronectin blocking antibodies prior to the infection significantly reduced the *S. aureus* adhesion level to injured corneal epithelium (*p* < 0.001). Finally, *S. aureus* clinical isolates triggered its internalization in human corneal epithelial cells as efficiently as the 8325-4 wt.

**Conclusion:**

*S. aureus* was almost unable to bind the intact corneal epithelium, whereas a superficial epithelial injury of the corneal epithelium strongly increased *S. aureus* adhesion, which is mainly driven by the interaction between staphylococcal fibronectin-binding proteins and unmasked fibronectin molecules located underneath the most superficial layer of the corneal epithelium.

## Introduction

Bacterial keratitis (BK) is a potentially eye-threatening infection characterized by a loss of integrity of the corneal epithelium and an inflammation of the stroma. *Pseudomonas aeruginosa* and *Staphylococcus aureus* are the most common causes of BK in industrialized countries (Urwin et al., 2020) and belong to the ESKAPE pathogens group (acronym for *Enterococcus faecium*, *S. aureus*, *Klebsiella pneumoniae*, *Acinetobacter baumannii*, *P. aeruginosa*, and *Enterobacter* species defined by the Infectious Disease Society of America) because they are of particular concern considering their potential multidrug resistance mechanisms and virulence (Mulani et al., 2019).

*S. aureus* is both a life-threatening pathogen and a commensal that colonize the skin and the mucosa of approximately one-third of human beings (Verhoeven et al., 2014). Like many other bacterial species found in humans, *S. aureus* harbors a number of virulence factors, including adhesins capable of binding to receptors expressed at the surface of eukaryotic cells and extracellular matrix (ECM) molecules (Josse et al., 2017). Bacterial adhesins are important at the early stage of colonization and infection to let the bacteria attach to the host tissues.

The microbial surface component recognizing adhesive matrix molecules (MSCRAMMs) [e.g., fibronectin-binding protein A and B (FnBPA/B), clumping factor A and B (ClfA/B), collagen adhesin (CNA)] are major cell wall-anchored proteins that mediate the attachment to ECM such as collagen, fibrinogen, or fibronectin (Foster et al., 2013). *In vitro*, *S. aureus* is also known to be internalized by different kinds of non-professional phagocytic cells (NPPCs). The FnBP-Fn-α5β1 integrin pathway is widely acknowledged to be the main internalization process, but other *S. aureus* factors [e.g., autolysin, extracellular adherence protein (Eap), lipoprotein-like lipoproteins] can also trigger internalization but in a lesser extent.

Mechanisms involved in the very early stage of *S. aureus* keratitis have yet to be demonstrated. Experimental models of BK are needed to study how *S. aureus* could adhere and invade the corneal epithelium but also to develop both innovative preventive and curative strategies in the context of rising antibiotic resistance. However, relevant clinical models of BK with human tissue are often difficult to establish because of the highly limited availability of healthy corneas for research. *In vivo* models of BK in rabbit showed that an intact cornea is virtually impossible to infect, but these results are difficult to generalize to humans (Tang et al., 2012). Because the welfare of animals has become an important ethical issue, *ex vivo* models using human or animal corneas stored on agar support have been used to study the physiopathology of infections or to assess drug delivery methods but with only limited clinical relevance in humans (Pinnock et al., 2017; Ubani-Ukoma et al., 2019) mostly because of the lack of epithelial integrity and the presence of stromal edema. In human, factors that compromise the integrity of the epithelium such as contact lens wearing, ocular surface diseases, ocular trauma, or prior ocular surgery are known to increase the risk of BK (Green et al., 2008; Ng et al., 2015).

We developed an innovative active storage machine (ASM) that greatly improves the long-term storage of human corneal grafts (Garcin et al., 2019; Garcin et al., 2020) but also supports the regeneration of a cohesive multilayered epithelium (Garcin et al., 2020; Guindolet et al., 2021). We hypothesized that the factors that compromise the integrity of the corneal surface may facilitate the initiation of the infection.

In this study, we used human corneal explants stored in ASM to investigate molecular mechanisms involved during the early stage of *S. aureus* keratitis.

## Methods

### Bacterial Strains and Growth Conditions

Strains used in this study were listed in **Table 1**. Strains were cultured at 37°C on blood agar (43049, Biomérieux, Marcy l’Etoile, France) or into RPMIc medium consisting of RPMI-1640 (R8755, Sigma-Aldrich, Saint-Louis, MO, USA) supplemented with 10% heat-inactivated fetal bovine serum (S1810, Biowest, Nuaillé, France), 1× of non-essential amino acids (M7145, Sigma-Aldrich), 1 mM of L-glutamine (G7513, Sigma-Aldrich), 1 g/L of sodium bicarbonate (S8761, Sigma-Aldrich), and 5 mg/L of iron(II) sulfate (F7002, Sigma-Aldrich). All reagents used in this study are listed in **Supplementary Table S1**.

**Table 1:** Bacterial strains used in this study.

Strains	Relevant characteristics	Reference
8325-4	NCTC 8325 cured of prophages	(Novick, 1967)
8325-4 Δ*fnbA/B*	*fnb*A and *fnb*B isogenic mutant of 8325-4 (DU 5883)	(Greene et al., 1995)
SA113	NCTC 8325 derivative strain	(Weidenmaier et al., 2008)
SA113 Δ*srtA*	*srt*A isogenic mutant of SA113	(Weidenmaier et al., 2008)
SACOR001	Clinical isolate of keratitis	This study
SACOR002	Clinical isolate of keratitis	This study
SACOR003	Clinical isolate of keratitis	This study
SACOR004	Clinical isolate of keratitis	This study
SACOR005	Clinical isolate of keratitis	This study

### Immunofluorescence on Corneal Epithelial Cross Section

Corneas stored in ASM during 14 days were fixed in paraformaldehyde (PFA) 0.5% during 45 min, followed by inclusion in optimal cutting temperature compound (OCT, Sakura Finetek USA, Torrance, CA, USA) and storage at -20°C until use. Immunostaining was performed in 10-µm-thick frozen sections without prior permeabilization. Primary and secondary antibodies were all diluted at 1/500 and 1/1,000, respectively (see antibodies’ list in **Supplementary Table S2**). Nuclei were stained with TO-PRO-3 Iodide at 1 µM (T3605, ThermoFisher). Fluorescence was imaged using confocal scanning laser microscope FLUOVIEW (FV1200, Olympus, Tokyo, Japan).

### Measurement of *S. aureus* Adhesion to Fibronectin

A 96-well plate (Nunc MaxiSorp. 44-2404-21, Invitrogen, Waltham, MA, USA) was coated with 0.1 µg of human fibronectin (FC010, Merck, Darmstadt, Germany) per well at 4°C overnight. The wells were washed three times with sterile phosphate-buffered saline (PBS) and blocked with 2% bovine serum albumin (BSA; A3059, Sigma-Aldrich) and 2% goat serum (G9023-10ML, Sigma-Aldrich) for 2 h at 37°C. The wells were filled with 100 µl of *S. aureus* cells at 10^7^ CFU/ml in RPMIc or with 100 µl of sterile RPMIc for negative control, and the plate was incubated for 30 min at 37°C. The wells were washed three times with sterile PBS to remove non-adherent bacteria and fixed with PFA 1%. Bound *S. aureus* cells were labeled with anti-SPA primary antibody at 10 µg/ml (PA1-7246, ThermoFisher Scientific, Waltham, MA, USA) and Alexa Fluor 488-conjugated anti-rabbit secondary antibody at 2 µg/ml (A-11034, ThermoFisher). Fluorescence intensity at 488 nm was measured with a microplate reader (Fluoroskan Ascent FL, ThermoFisher). The mean value of the negative control wells was used to subtract the background signal. Three independent experiments were performed with duplicate well for each condition.

### *S. aureus* Infection of Human Corneas

Six corneas from the eye banks of St-Etienne and Besançon (French Blood Center), which were considered not suitable for transplantation (i.e., endothelial cell density lower than 2,000 cells/mm²), were used in the study. Corneas were stored for 14 days in ASM to enable the epithelium recover and mature as previously described (Garcin et al., 2019; Garcin et al., 2020; Guindolet et al., 2021) (**Supplementary Figure S1**). After reepithelialization, corneas were dip-washed in sterile Dulbecco’s phosphate-buffered saline (DPBS) with CaCl_2_ and MgCl_2_ (14040091, ThermoFisher). Then, superficial linear epithelial injury was made with a 19½ gauge needle (301500, BD, NJ, USA). Each cornea was cut in half. One-half was put in DPBS with CaCl_2_ and MgCl_2_, and the other half was incubated with a mix of polyclonal anti-fibronectin antibodies (A0245, Agilent, Santa Clara, CA, USA; F3648, Sigma-Aldrich) at 5.25 and 1.5 µg/ml, respectively. Then, half cornea was cut in two, and each quarter was infected with a 150-µl volume of *S. aureus* suspension in DPBS with CaCl_2_ and MgCl_2_ with a slight agitation for 2 h at 37°C. The strains 8325-4 and 8325-4 Δ*fnbA/B* were used at 10^8^ CFU/ml; the strains SA113 and SA113 Δ*srtA* were used at 10^7^ CFU/ml. To avoid bias due to potential variability between corneas, each strain pairs were used to infect the same cornea (one quarter with anti-fibronectin, the other without). After the infection step, corneas were dip-washed in DPBS with CaCl_2_ and MgCl_2_ and fixed in 0.5% PFA for 30 min. Next, the cornea quarters were incubated for 1 h at 37°C with a mouse anti-SPA antibody at 6.4 µg/ml (37644, Abcam, Cambridge, UK) and a mix of two rabbit polyclonal anti-fibronectin antibodies (A0245, Agilent; F3648, Sigma-Aldrich) at 5.25 and 1.5 µg/ml, respectively. The cornea quarters were dip-washed in DPBS with CaCl_2_ and MgCl_2_ and labeled with Alexa Fluor 488-conjugated goat anti-mouse secondary antibody (A32723, ThermoFisher) at 4 µg/ml, Alexa Fluor 555-conjugated goat anti-rabbit secondary antibody (A21428, ThermoFisher) at 4 µg/ml, and TO-PRO-3 (T3605, Invitrogen) at 0.671 µg/ml for 1 h at 37°C. The cornea quarters were then dip-washed in DPBS with CaCl_2_ and MgCl_2_ and flat-mounted between slide and coverslip with Vectashield (H-1000, Vector Laboratories, Burlingame, UK) to be observed by confocal laser scanning microscopy (CLSM) (FV1200, Olympus). Images were analyzed with the Fiji software to detect the area filled by unmasked fibronectin and *S. aureus* bound to cornea and to quantify the mean fluorescence intensity (MFI) of *S. aureus* bound to cornea (Schindelin et al., 2012) (see **Supplementary Material** for details). *S. aureus* adhesion level to injured cornea was normalized according to the area of unmasked fibronectin. *S. aureus* adhesion levels for each strain couple were normalized in reference to the wild type-related strain. Three independent experiments were performed in which five fields were imaged for each condition tested.

### Internalization Assay of Human Corneal Epithelial Cells

HCE-2 cells were seeded in 24-well plate at a density of 1.10^5^ cells/well and incubated at 37°C under 5% CO_2_ in Dulbecco’s modified Eagle’s medium (DMEM) Glutamax (10566016, ThermoFisher) supplemented with 5% of heat-inactivated fetal bovine serum (S8150, Biowest), 35% of F12-HAM nutrient mix (N4888, Sigma-Aldrich), 1% of antibiotic and antimycotic (A5955, Sigma Aldrich), and 0.025% of human epidermal growth factor (EGF; 85570C, Sigma-Aldrich) until cells reached confluence. Lysostaphin protection assay was performed as previously described (Rigaill et al., 2018). Briefly, 24 h prior to infection, the culture medium was replaced by RPMIc. HCE-2 cells were inoculated with *S. aureus* cells at a multiplicity of infection (MOI) of 1 for 8325-4 and 8325-4 Δ*fnbA/B* strains and an MOI of 0.1 for SA113 and SA113 Δ*srtA* as well as for clinical strains. Bacterial loads were quantified by using an automatic plater and a colony counter (Rigaill et al., 2018). Three independent experiments were performed with duplicate well for each condition.

For internalized *S. aureus* visualization on the corneal epithelium, the same infection method on cornea as in the previous paragraph was used, cornea was then fixed in 0.5% PFA for 45 min, then permeabilized with 0.1% Triton X-100 (9036-19-5, Sigma Aldrich) during 15 min and immunolabeled with anti-SPA antibody at 6.4 µg/ml (37644, Abcam) for 2 h at 37°C; the cornea was rinsed three times in DPBS, then labeled with AlexaFluor 488-conjugated anti-mouse secondary antibody (A21428, ThermoFisher), anti-CK12 (PHDH1, Cytoskeleton Inc., Denver, Colorado, USA), and TO-PRO-3 (T3605, Invitrogen) at 0.671 µg/ml for 1 h at 37°C. After three dip-washes in DPBS, cornea was flat mounted between slide and coverslip for CLSM observations.

### Ethics Statement

Corneal explants used in this study were obtained after informed consent of the relatives, as authorized by French bioethics laws. All procedures conformed to the tenets of the Declaration of Helsinki for biomedical research involving human subjects.

### Statistical Analysis

All statistical tests were performed with GraphPad Prism version 9.1.0 (GraphPad Software, San Diego, CA, USA). Values of MFI, areas, and bacterial loads were compared by using unpaired t-test for data sets with only two groups or by using one-way ANOVA with recommended *post-hoc* correction (Tukey’s or Dunnett’s correction) for data sets with multiple groups.

## Results

### Superficial Epithelial Injury Unmasked Fibronectin and Other Molecules

**Figure 1:**
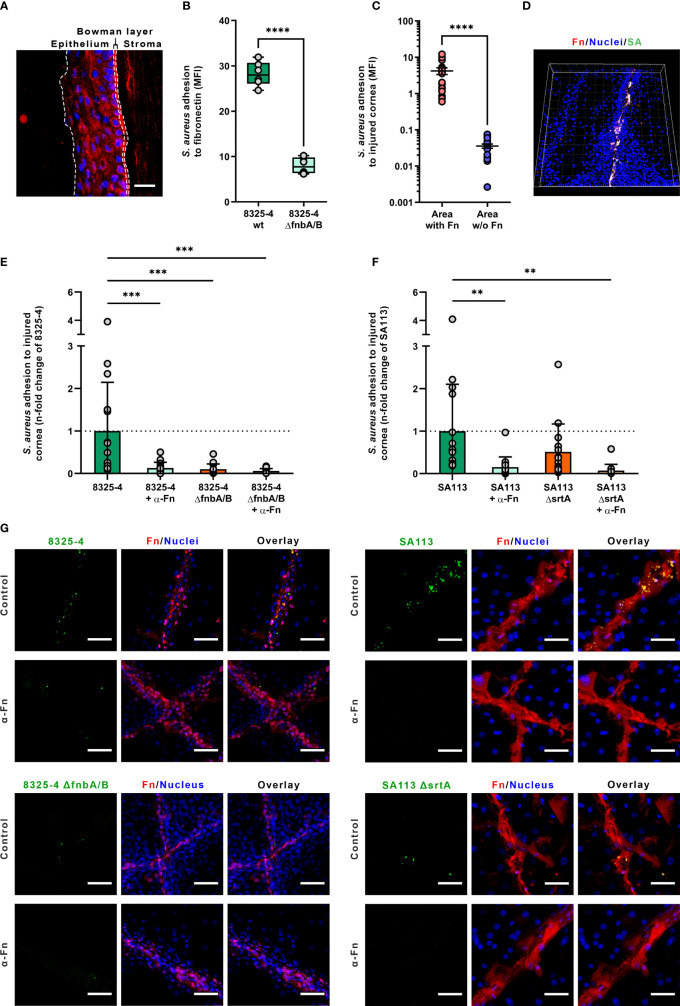
*Staphylococcus aureus* adhesion to human corneas. **(A)** Fibronectin immunolabeling of cryosection of human cornea. Dotted lines showed the limits between the epithelium, the Bowman’s layer and the stroma (nuclei in blue; fibronectin in red). Scale bar: 50 µm. **(B)** *S. aureus* adhesion to immobilized fibronectin. Dots represent all recorded values (three independent experiments, two wells per experiment). Box plots represent the mean and the standard deviation values. **(C)** *S. aureus* colocalization with fibronectin on human injured corneal epithelium. Dots represent the colocalization values of *S. aureus* cells for each recorded field. Bars represent the mean value with the standard deviation values. **(D)** Three-dimensional visualization of a corneal epithelium injured and infected with *S. aureus* 8325-4 wild-type (wt) strain (SA). **(E–G)** *S. aureus* adhesion level to injured corneal explants measured by confocal laser scanning microscopy. Dots represent *S. aureus* adhesion values normalized in reference to the 8325-4 strain **(E)** or the SA113 strain **(F)**. Images of human cornea explants infected with *S. aureus* are representatives of three independent experiments. The α-Fn condition corresponds to corneas incubated with anti-fibronectin antibodies prior to infection (see *Methods* for details). Scale bar: 50 µm **(G)**. MFI, mean fluorescence intensity; Fn, fibronectin; SA, *S. aureus*.

The localization of several molecules that could be targeted by *S. aureus* inside the corneal tissue was analyzed by immunofluorescence. Collagen I and IV were found only inside the stroma; laminin and CK5/6 were found to be distributed between cells close to the Bowman’s layer; involucrin, CK12, and β1 integrin (CD29) were expressed in the whole epithelium (**Supplementary Figure S2**). Fibronectin was found to be the most expressed ECM component in the whole epithelium but not at the epithelium surface (**Figure 1A**). Interestingly, a very superficial linear injury of the corneal epithelium was enough to unmask fibronectin molecules (**Figure 1D**).

### *S. aureus* Bound to Fibronectin Unmasked by Superficial Corneal Injury

*S. aureus* strains 8325-4 and 8325-4 Δ*fnbA/B* were used to assess fibronectin binding to injured corneal epithelium. First, we confirmed the fibronectin-binding capability of these two strains in static condition using immobilized fibronectin in microplate (**Figure 1B**). As expected, the deletion of *fnbA/B* genes decreased by 71% the adhesion level to immobilized fibronectin (bacterial MFI of 28.76 vs. 8.38 for *S. aureus* strains 8325-4 and 8325-4 Δ*fnbA/B*; *p* < 0.001).

On injured corneal tissue, *S. aureus* adhesion level was 117-fold increased where the corneal epithelium was injured (bacterial MFI of 0.04 vs. 4.21 in healthy and injured area, respectively; *p* < 0.0001) (**Figures 1C, D**). Interestingly, *S. aureus* 8325-4 was not able to efficiently bind intact corneal epithelium. By contrast, *S. aureus* 8325-4 Δ*fnbA/B* was not able to bind efficiently neither the injured nor the intact corneal epithelium (**Figure 1G** and **Supplementary Figure S3**).

To determine whether fibronectin exposure and staphylococcal FnBPs could play a role in the invasion of the corneal epithelium, we used anti-fibronectin blocking antibody to challenge injured cornea tissues with *S. aureus* strains lacking *fnbA/B* or *srtA* genes. The results showed that the blockage of fibronectin molecules significantly reduced the adhesion levels of *S. aureus* strains except for the 8325-4 Δ*fnbA/B*, which was nearly unable to bind fibronectin in any case (**Figures 1E, F**), demonstrating the major role of FnBPs for the adhesion to injured corneal epithelium.

With the SA113 strain, the deletion of the *srtA* gene, which is involved in the anchorage of cell wall proteins such as FnBPs, was found to decrease only slightly the adhesion level to injured corneal epithelium, suggesting that FnBPs are still anchored to the cell wall but probably less efficiently (**Figure 1G** and **Supplementary Figure S4**).

### Corneal Epithelial Cells Internalized *S. aureus* Clinical Isolates Recovered From Keratitis

**Figure 2:**
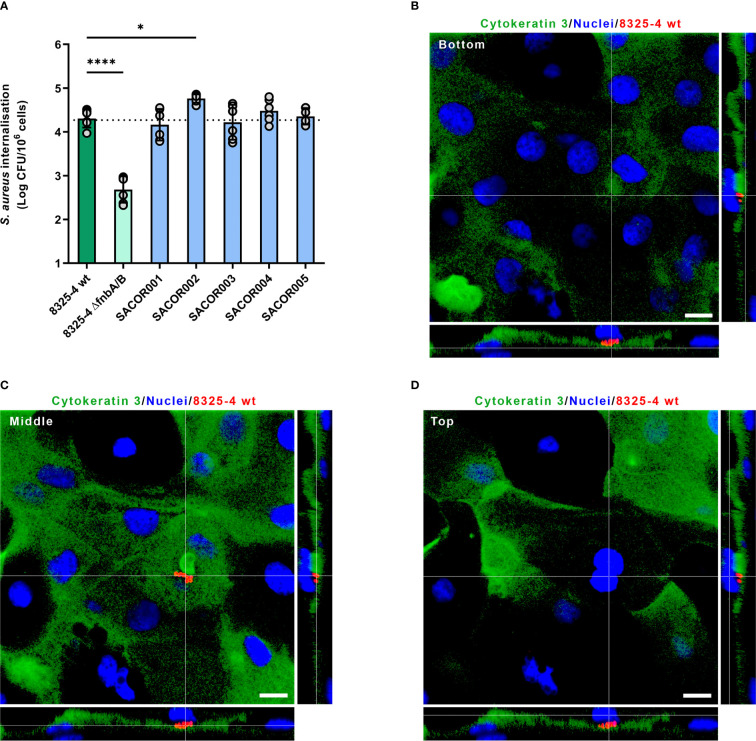
*Staphylococcus aureus* internalization inside corneal epithelial cells and human cornea. **(A)** Invasion of HCE-2 corneal epithelial cells by *S. aureus* clinical isolates recovered from patients with keratitis. Dotted line corresponds to the mean of intracellular load of *S. aureus* 8325-4 wild type (wt). **(B–D)** Confocal laser scanning microscopy of human cornea infected with *S. aureus* 8325-4. *S. aureus* cells (red) were located inside corneal epithelial cells (cytokeratin 3 immunolabeled in green, nuclei stained in blue). Orthogonal views correspond to a slice below *S. aureus* cluster (**B**; bottom), a slice within *S. aureus* cluster (**C**; middle), and a slice above *S. aureus* cluster (**D**; top).

Since the tripartite interaction between FnBPs expressed by *S. aureus*, fibronectin molecules, and the α5β1 integrin expressed at the surface of eukaryotic cells is acknowledged as the major pathway driving the internalization of *S. aureus* in NPPCs, we decided to investigate if *S. aureus* clinical isolate strains recovered from keratitis could trigger their internalization by corneal epithelial cells. Immunolabeling of HCE-2 cells showed that fibronectin molecules are detected at the cell surface (**Supplementary Figure S5**). We used the so-called lysostaphin protection assay with a monolayer of HCE-2, which is a cell line of human corneal epithelial cells, to assess the level of internalization of clinical isolates. All tested clinical isolates were internalized by HCE-2 cells and the 8325-4 strain (**Figure 2A**). One clinical isolate was internalized by HCE-2 cells significantly better than the 8325-4 strain; the intracellular loads were 4.3 ± 0.2 and 4.8 ± 0.1 log(CFU)/10^6^ cells for SARCOR002 and 8325-4 strains, respectively (*p* < 0.05).

Primary human corneal epithelial cells containing *S. aureus* cells were also observed by CLSM in injured corneas infected with *S. aureus* 8325-4 strain (**Figure 2B** and **Supplementary Figure S6**), which suggests that the tripartite interaction between FnBPs, fibronectin, and α5β1 integrin likely occurred during corneal infection *ex vivo*.

## Discussion

In this study, we used an *ex vivo* model of *S. aureus* infection of human corneas to elucidate the mechanisms involved in *S. aureus* adhesion to the corneal epithelium during the early stage of infection. We found that a superficial lesion is essential for *S. aureus* to adhere to the epithelium. Fibronectin was observed inside the corneal epithelium on cryosection, and we demonstrated that a superficial injury of the epithelium unmasks a large quantity of fibronectin molecules located just under the most superficial layer of the epithelium. However, we cannot exclude that injury induced the production of a small additional amount of fibronectin. We also showed that unmasked fibronectin molecules are targeted by FnBPs that enable the adhesion of *S. aureus*.

The tripartite interaction between human fibronectin present in ECM, α5β1 integrin expressed at the basolateral side of epithelial cells, and the *S. aureus* FnBPs is widely acknowledged as the main internalization pathway in NPPCs (Josse et al., 2017). However, clinical implications of this mechanism are still misunderstood. Here, we confirmed that FnBPs are essential adhesins for *S. aureus* to invade human corneal cells originating from an immortalized cell line as previously reported (Jett and Gilmore, 2002). Moreover, we evidenced that *S. aureus* is internalized by epithelial cells of the human cornea, suggesting that this bacterium could invade corneal cells during keratitis in humans. This finding is consistent with the fact that we and others have observed *S. aureus* inside human cells taken in healthy nasal carriers (Hanssen et al., 2017; Rigaill et al., 2018) and infected patients (Yang et al., 2018). Interestingly, we found that an intact corneal epithelium is highly resistant to *S. aureus* infection as described in animal models (Marquart, 2011; O’Callaghan, 2018).

To the best of our knowledge, this is the first study on *S. aureus* BK aimed at investigating the mechanisms of bacterial attachment to the epithelium surface using an innovative *ex vivo* model of human cornea infection. By using the ASM developed by the BiiGC laboratory, we were able to regenerate the human corneal epithelium to overcome the scarcity of human corneas for research purposes. The characteristics of the corneal epithelium regenerated after storage in the ASM are very similar to those observed for freshly collected corneas (Guindolet et al., 2021). This model allows studying the molecular interactions between *S. aureus* and human primary epithelial cells physiologically polarized within a cornea, which is highly clinically relevant to identify which cell host receptors are reachable by pathogens. The model of BK we developed is based on a superficial lesion of cornea that only disrupts the outer layer of the epithelium. This technique seems to be more clinically relevant than large injury (that can damage the Bowman’s layer) required to establish BK in animals (Marquart, 2011; O’Callaghan, 2018). Further improvements are ongoing to establish BK inside the ASM using air lifting mode in order to assess if the eyelid blink could play a role for the clearance of infection (Guindolet et al., 2021). As previously developed to study *S. aureus* nasal colonization (Krismer et al., 2014), a synthetic medium mimicking the chemical properties and the innate immune activity of tear drops could help to better understand both bacterial and human factors that support the onset of BK.

We demonstrated that a very superficial injury of the cornea dramatically increases the *S. aureus* invasion of the human corneal epithelium. These findings support the clinical evidence that contact lens wear and a small eye trauma or surgery are well-recognized risk factors of BK (Ng et al., 2015; O’Callaghan, 2018). In our model, *S. aureus* wild-type strains were able to strongly bind to the human corneal epithelium only when the outer cell layer is injured. By using mutant strains and blocking antibodies, we evidenced that binding and invasion of the human cornea are mainly driven by FnBPs, but we cannot totally exclude that other staphylococcal adhesins play a small role when FnBPs are lacking. However, the *fnbA/B* genes coding for FnBPs are carried by the vast majority of clinical strains (Peacock et al., 2000) and by all keratitis isolates tested in this study, suggesting that these adhesins are essential for establishing keratitis in humans but also for colonizing and infecting humans more generally. Interestingly, the SA113 strain deleted for the *srt*A gene, which is involved in the anchorage of cell wall proteins such as FnBPs, was found to only slightly decrease the adhesion level to injured corneal epithelium, suggesting that either FnBPs are still anchored [but less efficiently as previously described in (Weiss et al., 2004)] or another fibronectin-dependent mechanism is used by this strain. Several studies showed that both secreted and cell wall-anchored proteins such as clumping factor A/B, iron-regulated surface determinant A, *S. aureus* surface protein G, or extracellular adherence protein can bind fibronectin molecules (Foster et al., 2013). Hume et al. (2020) reported that staphopain A secreted by *S. aureus* promotes the bacterial adhesion in a mouse model of keratitis using the clinical isolate Staph 38 recovered from a human corneal ulcer but not with the NCTC 8325 strain initially isolated from a human corneal ulcer. In our study, all the clinical isolates included had roughly the same capability to invade corneal epithelial cells than the 8325-4 strain, which is indeed the 8325 strain curated of its three prophages for laboratory use (Novick, 1967). Further studies are needed to unravel how staphopain A could be involved in *S. aureus* keratitis in humans.

In a clinical point of view, we provided evidence that *S. aureus* is able to invade human epithelial cells located inside the cornea epithelium. This observation should encourage the use of antibiotics able to target *S. aureus* inside host cells such as fluoroquinolone or rifampicin (Valour et al., 2015; Rigaill et al., 2018). In both methicillin-susceptible *S. aureus* (MSSA) and methicillin-resistant *S. aureus* (MRSA), the increase of resistance to fluoroquinolones, which is the first line of treatment of BK (Urwin et al., 2020), may reduce the possibility of treating intracellular bacteria effectively.

This study highlights the main role of fibronectin adhesion at the early stage of BK in humans. These findings should encourage research into molecules that limit bacterial adhesion, especially in situations where the risk of infection is increased (e.g., trauma, surgery). Further studies are needed to assess if eye drops containing molecules able to mask fibronectin motifs targeted by bacteria could be used to prevent BK. In addition, it is important that effective treatments for intracellular bacteria be researched and improved (Abad et al., 2021). Finally, whether *S. aureus* carriage can increase the risk of endogenous *S. aureus* keratitis remains unknown probably because of a relatively low incidence as compared to other kinds of *S. aureus* infections (Verhoeven et al., 2014). Nevertheless, *S. aureus* nasal carriage is a well-established risk factor of endogenous infection in a broad range of clinical settings (Verhoeven et al., 2014). It could be considered to develop effective strategies to efficiently prevent BK due to *S. aureus*.

## Data Availability Statement

The original contributions presented in the study are included in the article/**Supplementary Material**. Further inquiries can be directed to the corresponding author.

## Ethics Statement

Ethical review and approval were not required for the study on human participants in accordance with the local legislation and institutional requirements. Written informed consent for participation was not required for this study in accordance with the national legislation and the institutional requirements.

## Author Contributions

Conceptualization: CM, EC, ZH, PG, GT, and PV. Investigation: CM, EC, ZH, JR, JJ, FL, PG, GT, and PV. Methodology: CM, EC, ZH, JR, and PV. Supervision: PG, GT, and PV. Writing-original draft: CM and PV. Writing—review and editing: ZH, JJ, FL, PG, GT, and PV. All authors contributed to the article and approved the submitted version.

## Conflict of Interest

The authors declare that the research was conducted in the absence of any commercial or financial relationships that could be construed as a potential conflict of interest.

## Publisher’s Note

All claims expressed in this article are solely those of the authors and do not necessarily represent those of their affiliated organizations, or those of the publisher, the editors and the reviewers. Any product that may be evaluated in this article, or claim that may be made by its manufacturer, is not guaranteed or endorsed by the publisher.
