# Supplementary material for: Key Role of Staphylococcal Fibronectin-Binding Proteins During the Initial Stage of Staphylococcus aureus Keratitis in Humans
Source: Front Cell Infect Microbiol. 2021 Nov 9;11:745659. doi: 10.3389/fcimb.2021.745659 (PMC8630648; doi:10.3389/fcimb.2021.745659)
Supplement: Supplementary file 1 [file DataSheet_1.pdf]

Supplementary Material

Supplementary Tables

Supplementary Table 1. Antibodies used in this study

Primary antibodies			
**Target Protein**	**Isotype**	**Source**	**Reference**
Fibronectin	Polyclonal	Rabbit	F3648; Sigma-Aldrich
Fibronectin	Polyclonal	Rabbit	A024502-2; Agilent
Staphylococcus aureus	Polyclonal IgG	Rabbit	PA1-7246; Invitrogen
Staphylococcal Protein A	Monoclonal IgG3	Mouse	Ab37644; Abcam
β1 Integrin/CD29	Monoclonal IgG2a	Mouse	Sc59829; SCBT
Collagen I	Polyclonal IgG	Rabbit	Ab34710; Abcam
Collagen IV	Monoclonal IgG	Mouse	Ab6311; Abcam
Cytokeratin 10	Monoclonal IgG1	Mouse	MA1-06319; Invitrogen
Cytokeratin 5 + 6	Monoclonal IgG1	Mouse	Ab17133; Abcam
Cytokeratin 12	Monoclonal IgG2b	Mouse	Sc515882; SCBT
Involucrin	Monoclonal IgG	Mouse	I9018; Sigma-Aldrich
Laminin V	Monoclonal IgG	Mouse	Sc13586; SCBT
**Secondary antibodies**			
**Target IgG**	**Fluorophore**	**Purification method**	**Reference**
Goat anti-rabbit IgG	Alexa Fluor 555	Highly cross-adsorbed	A21428; Invitrogen
Goat anti-mouse IgG	Alexa Fluor 488	Highly cross-adsorbed	A32723; Invitrogen
Goat anti-rabbit IgG	Alexa Fluor 488	Highly cross-adsorbed	A11034; Invitrogen

Supplementary Table 2. Reagents used in this study

Reagent	Supplier	Reference
AMBICIN, recombinant lysostaphin	Ambi Products LLC	LSPN
HCE-2 cells	ATCC	CRL-11135
BD Microlance 3 19 ½ Gauge Needle	BD	301500
Columbia agar + 5% sheep blood	Biomérieux	43049
Fetal Bovine Serum	Biowest	S1810
96-well plate (Nunc MaxiSorp)	Invitrogen	44-2404-21
Human plasma fibronectin	Merck	FC010
Antibiotic, antimycotic solution (100x)	Merck	A5955
LONG EGF human	Merck	85570C
RPMI-1640	Sigma-Aldrich	R8755
Non-essential amino acids	Sigma-Aldrich	M7145
L-Glutamine	Sigma-Aldrich	G7513
Sodium bicarbonate	Sigma-Aldrich	S8761
Iron(II) Sulphate	Sigma-Aldrich	F7002
Bovine serum albumin	Sigma-Aldrich	A3059
Goat serum	Sigma-Aldrich	G9023
F12-HAM nutrient mix	Sigma-Aldrich	N4888
DPBS with $CaCl_2$ and $MgCl_2$, sterile	ThermoFisher	14040091
DMEM Glutamax	ThermoFisher	10566016
Mounting media	Vector laboratories	H-1000
TO-PRO-3 Iodide	ThermoFisher	T3605

Supplementary Figures

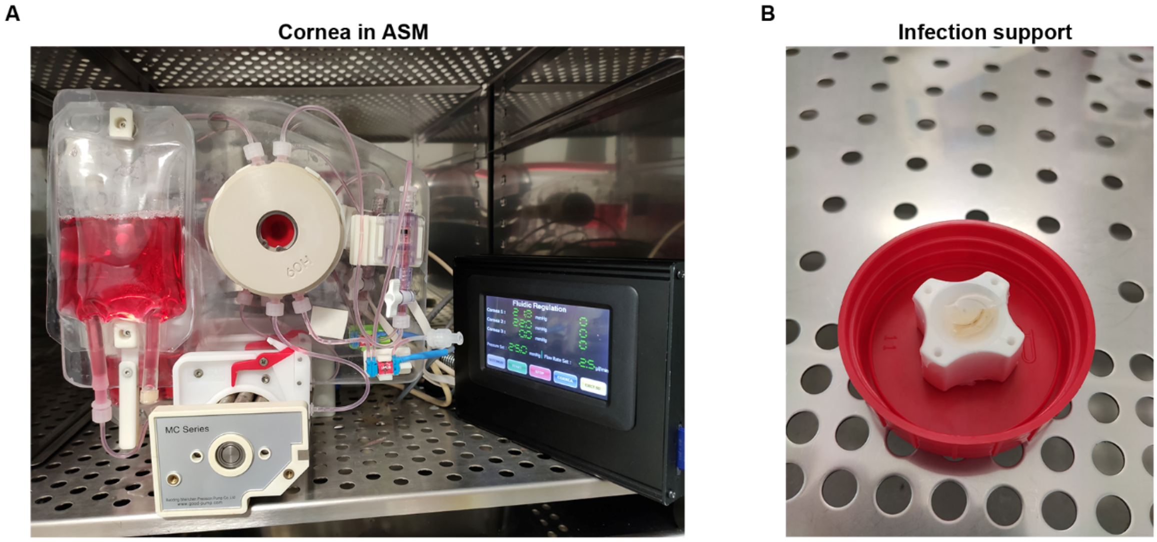

Supplementary Figure 1. Human cornea prior to *S. aureus* infection. (A) Cornea stored in ASM (Active Storage Machine) regulated with a 21-22 mmHg of endothelial pressure and a 2,6 µL/min flow rate. **(B)** Quarter of cornea seated on the infection support.

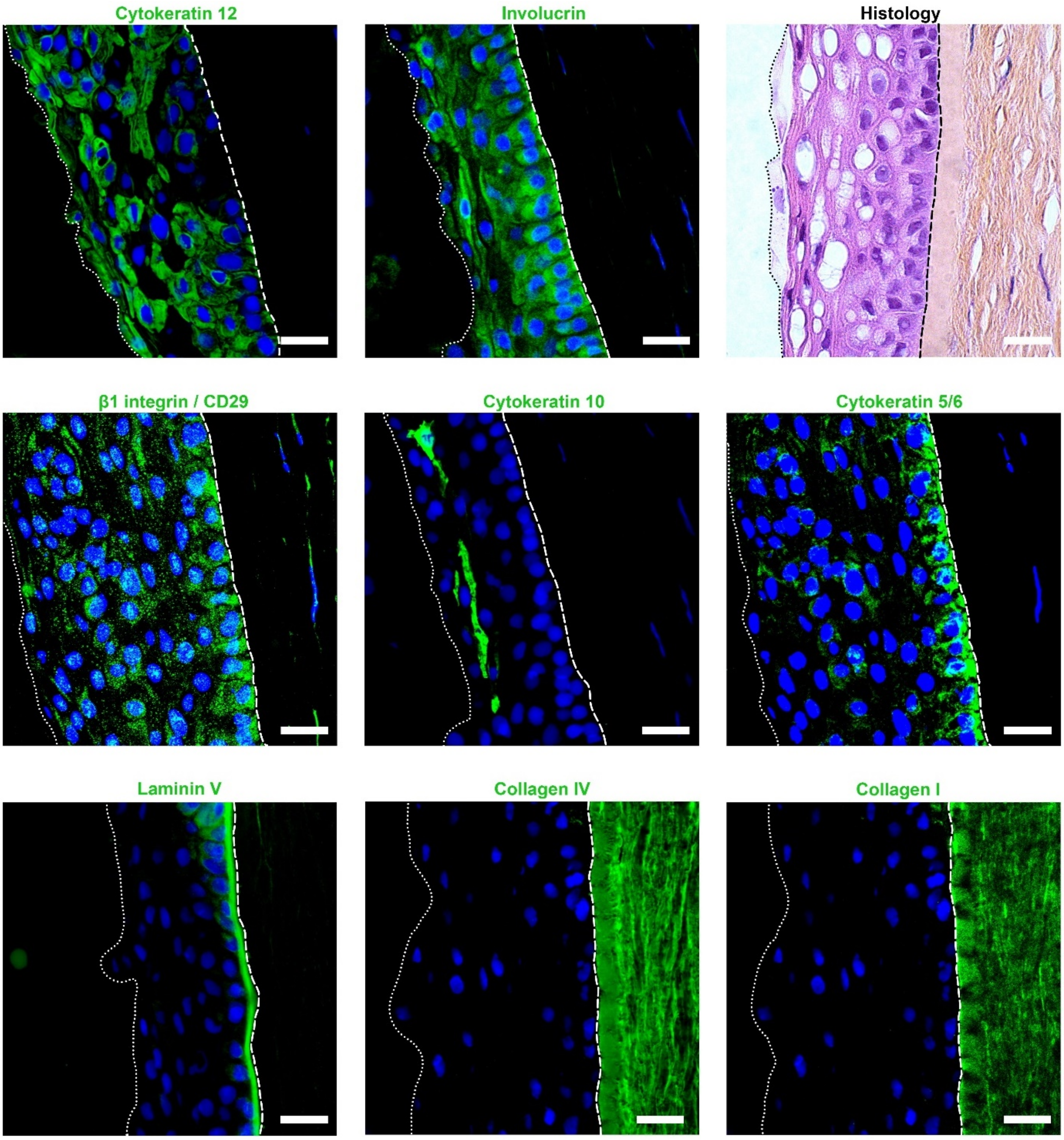

Supplementary Figure 2. Confocal laser scanning microscopy of cross sections of human corneas re-epithelialized 14 days in active storage machine, Dotted lines correspond to the air/epithelium interface, Dashed lines correspond to the epithelium/Bowman separation. Scale bar: 30 µm. (target receptor in green, nuclei in blue).

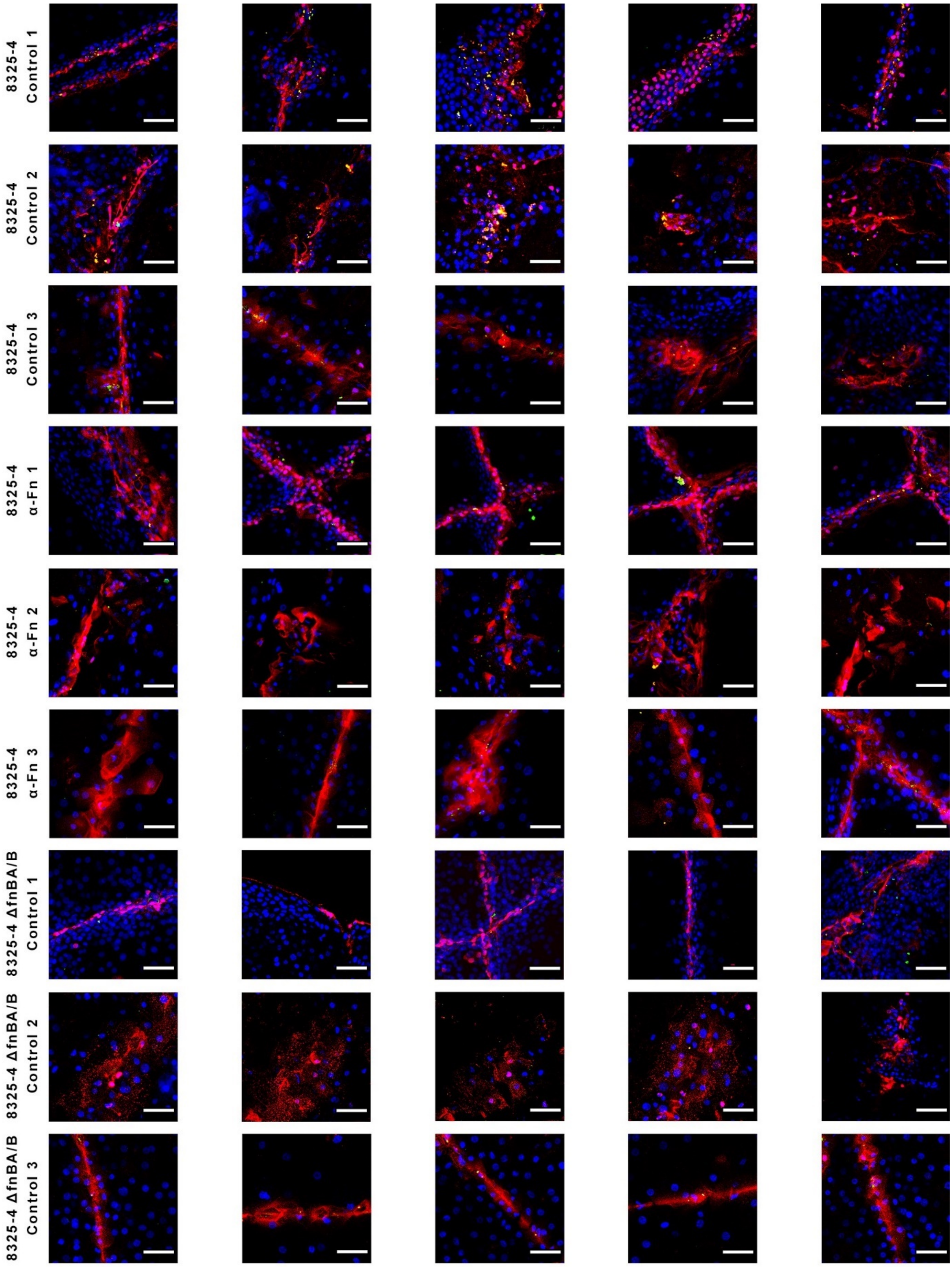

Supplementary Figure 3. Injured cornea infected with *S. aureus* 8325-4 related strains (continued on next page).

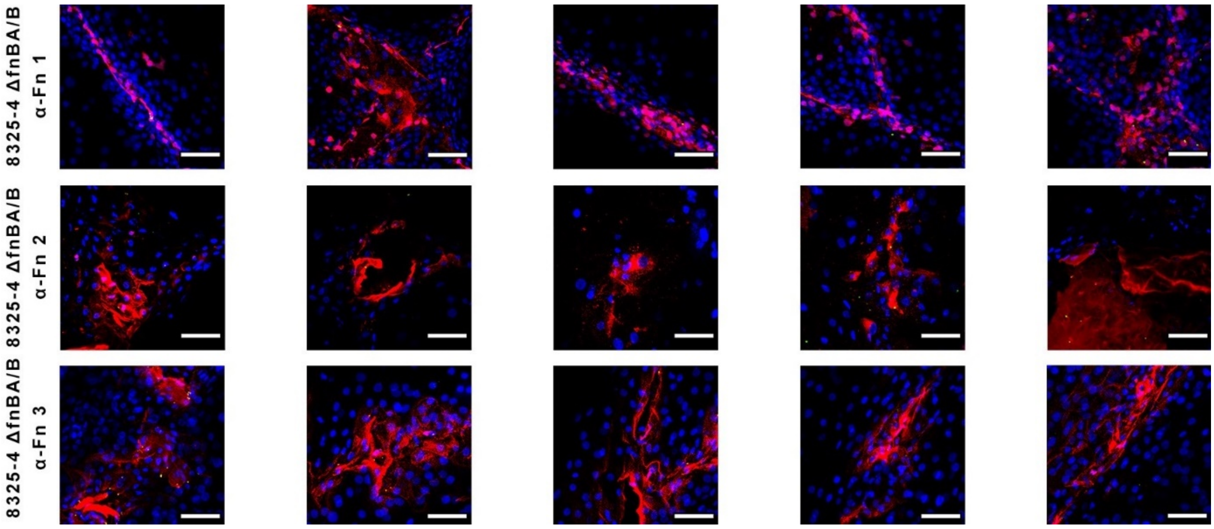

Supplementary Figure 3. Injured cornea infected with *S. aureus* 8325-4 related strains (continued). Images represent Z-stack projections of images acquired by confocal laser scanning microscopy. The α-Fn condition corresponds to explants incubated with anti-fibronectin antibodies prior to infection (see methods for details). Nuclei are depicted in blue. Fibronectin molecules are depicted in red. Fn: Fibronectin.

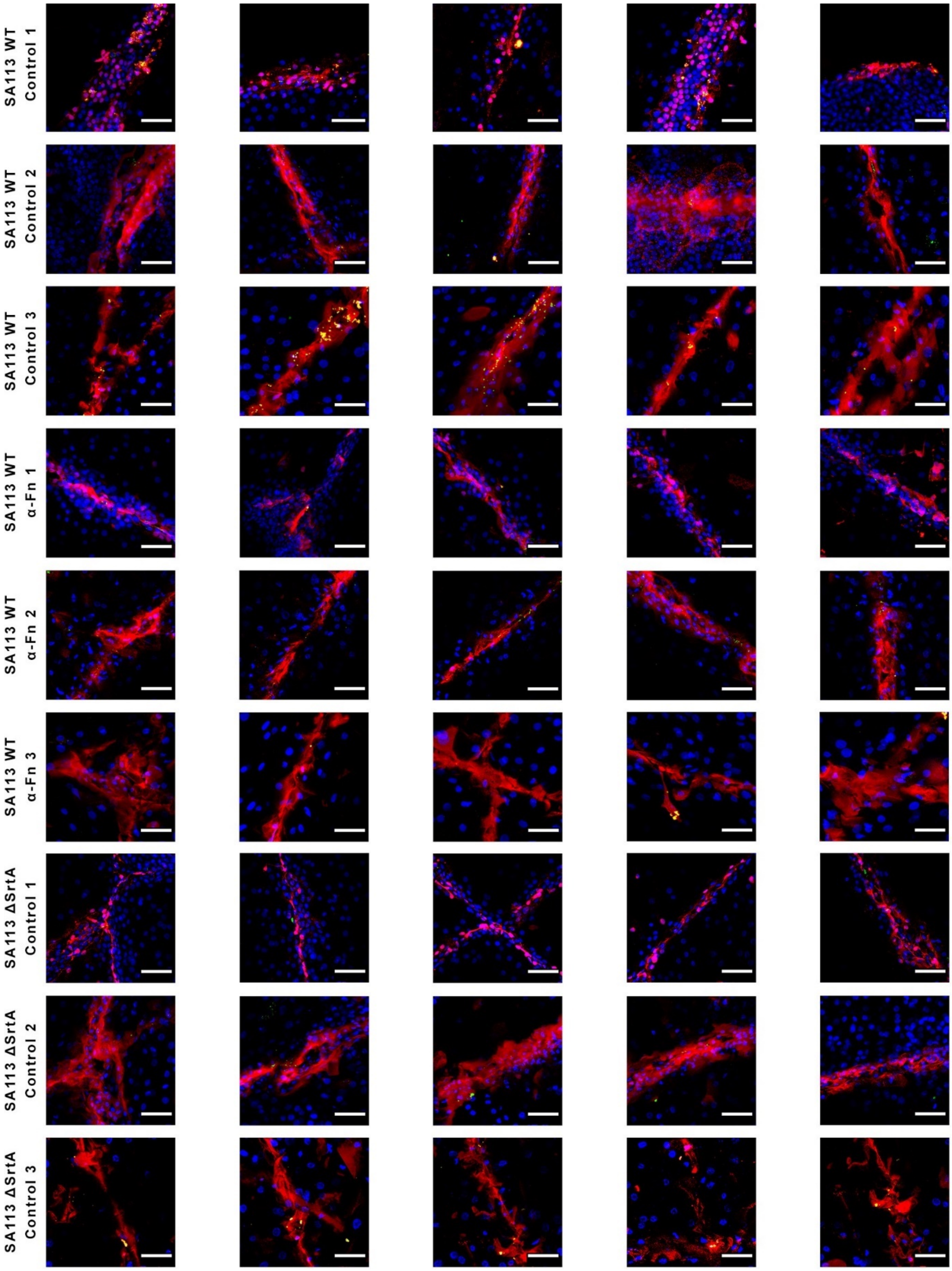

Supplementary Figure 4. Injured cornea infected with *S. aureus* SA113 related strains (continued on next page).

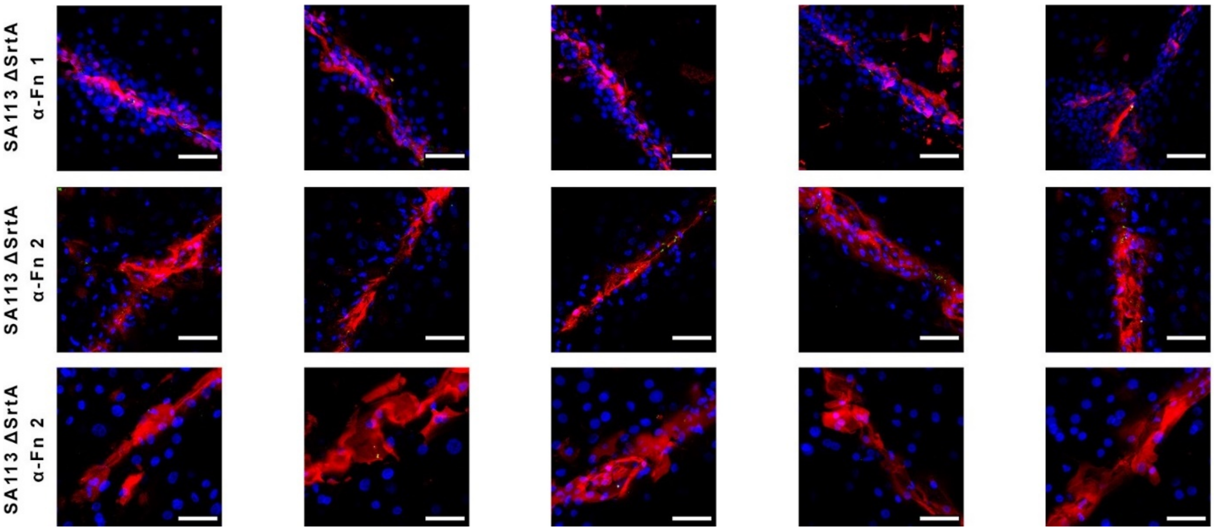

Supplementary Figure 4. Injured cornea infected with *S. aureus* SA113 related strains (continued). Images represent Z-stack projections of images acquired by confocal laser scanning microscopy. The α-Fn condition corresponds to explants incubated with anti-fibronectin antibodies prior to infection (see methods for details). Nuclei are depicted in blue. Fibronectin molecules are depicted in red. Fn: Fibronectin.

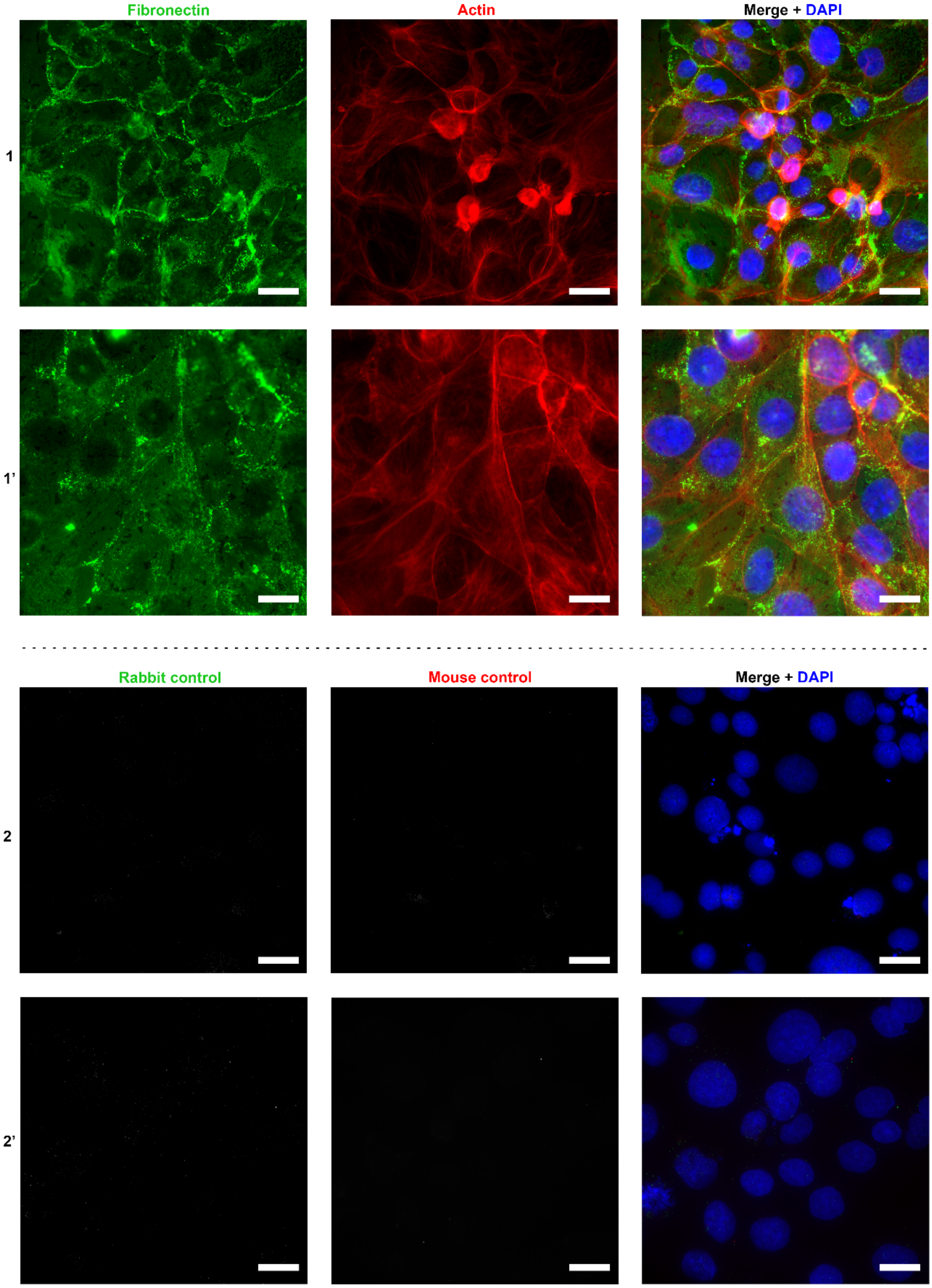

Supplementary Figure 5. Epifluorescence microscopy of immortalized human corneal epithelial cells (HCE-2). Pictures 1 (x600, scale bar: 25µm) and 1' (x1000, scale bar: 15 µm) showed immunolabelling targeting fibronectin (green) and actin (red). Pictures 2 (x600, scale bar: 25µm) and 2' (x1000, scale bar: 15 µm) showed immunolabelling targeting rabbit isotype antibody (green) and mouse isotype antibody (red). Nuclei were stained with DAPI (blue).

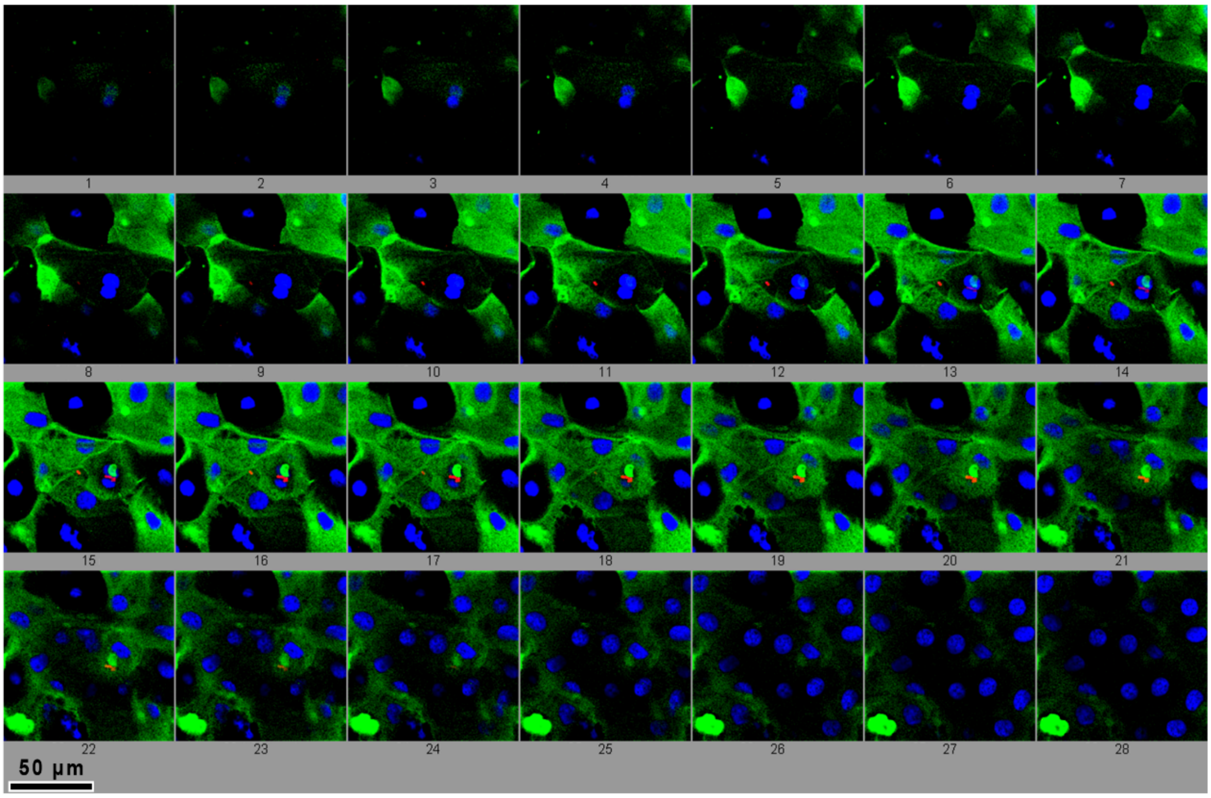

Supplementary Figure 6. Images show all the 28 slices (z-step of 0.6 µm) used in Figure 2B-D. Images acquired by confocal laser scanning microscopy show *S. aureus* (red) inside corneal epithelial cells *ex vivo* (cytokeratin 3 immunolabelled in green; nuclei stained in blue). The number indicated under each image corresponds to the slice's number.

Supplementary methods

Confocal images were analyzed with Fiji software (v1.52).

The area of unmasked fibronectin (Fn) was defined by the signal of Alexa Fluor 555 acquired on the 555 nm channel. Z-stack reduction was performed using max intensity projection (Stacks > Z project > max intensity). A median filter (radius: 2.0) was applied. The threshold value was set at 1702/4095 (default, B&W, dark background). The Fn area was measured using the “analyze particle” function (Analyze > Analyze particles > Size µm²: 0-infinity; Circularity: 0.00-1.00).

The mean fluorescence intensity (MFI) of *S. aureus* was defined by the fluorescence intensity of Alexa Fluor 488 acquired on the 488 nm channel. Z-stack reduction was performed using average intensity projection (Stacks > Z project > average intensity). The mean intensity of the GFP signal was measured inside or outside the Fn area (Analyze > Measure).

The *S. aureus* area was defined by the signal of Alexa Fluor 488 acquired on the 488 nm channel. Z-stack reduction was performed using max intensity projection (Stacks > Z project > max intensity). For the *S. aureus* 8325-4 related strains, no median filter was applied and the threshold was set to 1702. For the SA113 related strains, a median filter (Radius: 1.0) was applied and the threshold was set to 1244. The S. aureus area was measured using the “analyze particles” function (Analyze > Analyze particles > Size µm²: 0.5-infinity; Circularity: 0.00-1.00).
